# Necroptosis-related lncRNA signatures determine prognosis in breast cancer patients

**DOI:** 10.1038/s41598-022-15209-3

**Published:** 2022-07-04

**Authors:** Yuan Zhang, Qingfang Yue, Fei Cao, YanQin Li, Yifang Wei

**Affiliations:** 1 Department of Oncology, Shaanxi Provincial People’s Hospital, Xi’an, 710068 Shannxi China; 2 Xi’an Medical University, Xi’an, 710021 Shannxi China; 3 Center for Reproductive Medicine, Naval Medical Center, Second Military Medical University, 338 Huaihai West Road, Shanghai, 200052 China

**Keywords:** Breast cancer, Cancer models, Tumour biomarkers, Tumour heterogeneity, Tumour immunology, Necroptosis, Cell death and immune response, Immunotherapy, Inflammation

## Abstract

Necroptosis is a genetically regulated form of necrotic cell death that has emerged as an important pathway in cancers. Long non-coding RNAs (lncRNAs) are key regulators of breast cancer development. Nevertheless, few studies are reporting the effect of lncRNAs in necroptosis processes and the role of necroptosis-related lncRNAs (NRLs). The present study aimed to construct a prognostic model based on NRLs in breast cancer. NRLs were identified by combining expression profiling data from The Cancer Genome Atlas (TCGA) with necroptosis-related genes. The non-negative matrix factorization (NMF) clustering analysis was conducted to identify molecular subtypes of BC, and the clinical outcome and tumor-infiltrating immune cells (TIICs) in the different molecular subtypes were analyzed. Four molecular subtypes based on NRLs were identified, and these four molecular subtypes could predict clinical features, prognosis, and tumor-infiltrating immune cells (TIICs). A 4-NRLs signature and nomogram were established and validated its predictive capability of overall survival (OS) in breast cancer patients. Analyses of clinicopathological features, prognosis, TIICs, tumor microenvironment (TME), somatic mutations, and drug response revealed significant differences between the two risk groups. In addition, we found that low-risk patients exhibited higher levels of immune checkpoints and showed higher immunogenicity in immunophenoscore (IPS) analysis. In conclusion, we constructed a prognostic model based on the expression profile of NRLs, which may facilitate the assessment of patient prognosis, immunotherapeutic responses, and maybe a promising therapeutic target in clinical practice.

## Introduction

Breast cancer (BC) is one of the most frequently diagnosed cancers in women worldwide^1^. BC consists of several molecular subtypes and is a highly heterogeneous malignancy. The diversity between and within tumors and between individuals together determines BC prognosis and drug resistance^2^. Despite progress in treatment strategies, some patients still have poor prognosis, given the lack of effective screening tools and difficulties in early diagnosis and prognosis^3^. Therefore, new and accurate prognostic and diagnostic tools are needed to optimize targeted treatments.

In recent years, the traditional definition of cell death has begun to blur as a hybrid pattern of cell death that exhibits features of apoptosis and morphological features that overlap with necrosis (termed “aponecrosis”) has been gradually recognized^4,5^. Necroptosis is a new form of programmed necrotic cell death that is similar in mechanism to apoptosis and morphologically similar to necrosis^6^. The role of necroptosis in the progression of cancer is complicated. On the one hand, tumor cells can be eliminated directly by the process of necroptosis. In addition, necroptosis provides antigenic and inflammatory stimuli to dendritic cells to trigger a robust adaptive immune response that halts tumor progression^7^. On the other hand, recruited inflammatory responses may also promote tumorigenesis and cancer metastasis, while necroptosis may generate an immunosuppressive tumor microenvironment^7^.

Long non-coding RNAs (lncRNAs) are non-coding RNAs that are more than 200 nucleotides, and the majority of these RNAs do not have protein-coding ability^8^. However, increasing evidence has revealed the contributions of lncRNA in cancer phenotypes by physically interacting with proteins, DNA, and other RNA^8^. lncRNAs can regulate multiple biological processes, including tumorigenesis and immunity^9,10^. Previous studies have emphasized the effect of protein-coding genes in necroptosis execution^11–13^. However, to our best known, it remains to be elucidatedwhether and how non-coding RNAs regulate necroptosis sin BC, and the relationships between necroptosis-related lncRNAs (NRLs) with survival in BC patients have never been explored. Thus, the identification of NRLs is crucial for revealing the potential mechanism of BC and identifying new therapeutic targets. Recently, two lncRNA signatures associated with necroptosis were established in BC^14,15^. However, none of them explored the heterogeneity of BC, but simply used NLRs to construct a prognostic model. In the present study, for the first time, weidentified four molecular subtypes of BC based on NRLs and developed a signature for predicting the OS of BC patients. Meanwhile, we also investigated the correlations of the prognostic signature with clinical features, tumor immune microenvironment, somatic mutation landscapes, and chemosensitivity. Ultimately, nomograms were constructed and allowed for improved accuracy in survival estimation.

## Materials and methods

### Data extraction

RNA sequencing (RNA-seq) data (with FPKM values), including cancer group (n = 1104) and normal group (n = 292), and respective clinical properties of breast cancer were retrieved from TCGA database (https://portal.gdc.cancer.gov/) and Genotype-Tissue Expression (GTEx) databases^16^. For cleaning the TCGA data, this study eliminated samples with overall survival of < 30 days.

### Screening for necroptosis-related lncRNAs

A total of 67 necroptosis-related genes were obtained from the previous publication^17^. Then, Pearson’s correlation analysis was performed for necroptosis-related lncRNAs (NRLs) identified. LncRNAs with |Pearson R|> 0. 5 and p < 0.05 were considered to be NRLs.

### Consensus clustering based on NRLs

We performed a non-negative matrix factorization (NMF) clustering analysis with the “NMF” R package and clustered the samples into different groups^18^. The correlations between our NRLs subtypes, clinical traits, and prognosis were analyzed.

### Immune activities of the molecular subtypes

To understand the characteristics of immune cells in different subtypes, The CIBERSORT algorithm was employed to analyze the relative abundance of 22 kinds of TIICs in the immune microenvironment^19^.

### Establishment of NRLs signature

At a ratio of 1:1, all BC patients were classified as training and testing sets. Co-expressed NRLs were tested using the univariate Cox analysis to determine the prognostic value. Subsequently, based on the candidate lncRNAs with p < 0.05 in the univariate Cox screen analysis, a least absolute shrinkage and selection operator (LASSO) regression model was constructed to reduce the dimension of high latitude data using the R package “glmnet”^19^. Ten-fold cross-validation was employed to avoid the overfitting problem and select the penalty parameter (λ) according to the minimum criteria. The resulting lncRNAs were introduced in a multivariate Cox model to obtain the hazard ratio (HR) and the regression coefficient. Risk scores for BC patients were estimated below:

Risk Score = ${\sum }_{i=1}^{n}expi*coefi$, where exp and coef are expression levels and correlation coefficients, respectively. BC patients were divided into the high- and low-risk subgroups based on median risk score, and OS times were comparatively assessed in both subgroups using Kaplan–Meier curves. PCA and t-SNE were carried out for dimensionality reduction analysis to assess the ability to distinguish characteristics of patients at different risks. The receiver operating characteristic (ROC) curves and Harrell’s concordance index (C-index) were performed for assessment of the performance of the signature. Moreover, in the testing set, the same methods were adopted to examine the accuracy of the risk score model.

### Clinical correlation analysis and stratification analysis of signature

We performed a clinical correlation analysis between the risk score and clinicopathological features, including age, molecular subtype, TNM stage, surgery type, and margin status. In addition, the KM analysis was conducted to elucidate differences between two risk subgroups based on age (≤ 60 and > 60 years), molecular subtype (basal, HER2, luminal A, luminal B, and normal), stage (I, II, and III–IV), surgery type (lumpectomy, simple mastectomy, modified radical mastectomy), margin status (positive and negative).

### Immune infiltration and IPS analyses

CIBERSORT algorithm was employed to analyze the expression profile of BC patients to infer the relative proportion of the 22 types of tumor-infiltrating immune cells (TIICs)^20^. The Spearman correlation between the abundance of TIICs and CRG score was analyzed. The immune, stromal, and ESTIMATE scores for each sample were also analyzed. We also extracted potential immune checkpoints from previous literature, then compared and analyzed differences among them at p < 0.05. The immunophenoscore (IPS) computed a score based on the gene expression values of immune-related genes into four classes: (1) effector cells, (2) immunosuppressive cells, (3) MHC molecules, and (4) selected immunomodulators^21^. The IPS of BC patients were obtained from TCIA (https://tcia.at/).

### Mutation and drug sensitivity analyses

To compare the mutation load between the two risk groups, the mutation annotation format (MAF) from the TCGA database was accomplished by the “maftools” R package. Wilcoxon test was used to evaluate the difference between the somatic mutation and TMB levels between the two risk groups. To estimate the risk score in the clinical treatment of BC, we employed the “pRRophetic” package to calculate the TCGA project of the BC dataset for the half-maximal inhibitory concentration (IC50) of commonly used chemotherapeutic agents. The algorithm allows participants to apply baseline tumor gene expression profiles to predict clinical chemotherapy response, which is obtained by establishing statistical models from the gene expression and drug sensitivity data derived from cell lines in the Cancer Genome Project.

### Construction of a predictive nomogram

A nomogram for predicting OS in BC patients was established by combining the risk score with clinicopathological traits using the “rms” R package. ROC curves and calibration plots were employed to evaluate the predictive performance of the model.

### Functional analysis

To evaluate the possible biological functions of NRLs, GSEA was performed to detect the associated GO terms and KEGG pathways between the low- and high-risk groups. The Hallmarks, c5.go.v7.4 and C2 KEGG v.7.4, were selected, and the number of permutations was set to 1000 times. The outcomes that meet p < 0.05 was considered statistically significant.

### Statistical analysis

R software 4.1.0 (https://www.r-project.org/) was used in this research. Survival curves for prognostic analysis were generated by the Kaplan–Meier method, and the log-rank test was used to determine the significance of differences. ROC analysis was applied to verify the accuracy of the signature in predicting survival. Univariate and multivariate analyses using Cox regression helped us to determine the independent prognostic factors. P < 0.05 was used to determine statistical significance.

## Results

### Identification of NRLs in BC patients

A total of 67 necroptosis-related genes and 13,361 lncRNAs were identified via previous publications and RNA-seq data of BC patients, respectively. According to Pearson correlation analysis (correlation coefficient > 0.5 and P < 0.05), we finally obtained 360 NRLs (Tables S1, S2).

### Consensus clustering based on NRLs

**Figure 1:**
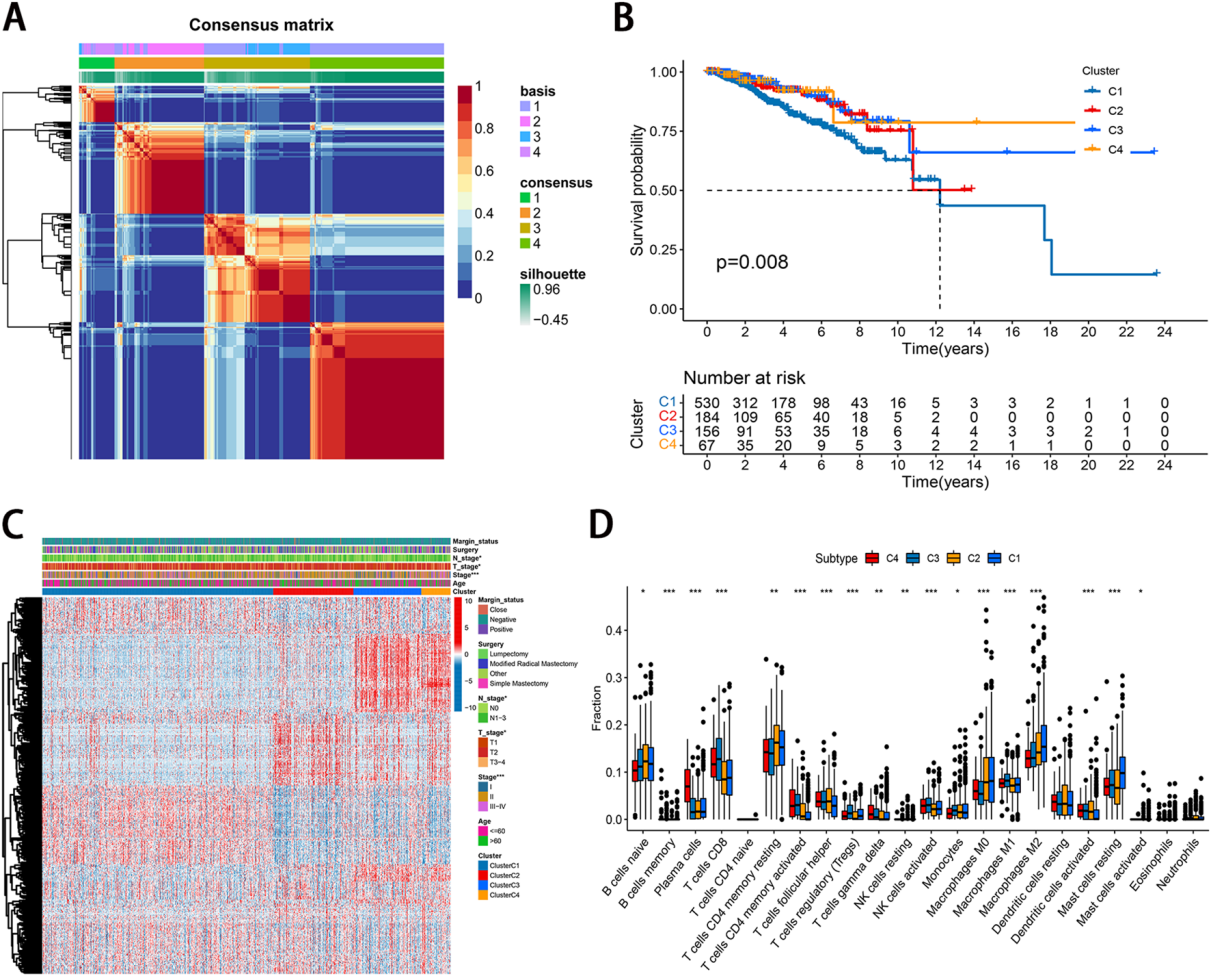
Identification molecular subtypes based on the necroptosis-related lncRNA. (**A**) NMF consensus clustering for the k value was 4. (**B**) Kaplan–Meier curves of OS for patients with the four subtypes. (**C**) Correlation between the four subtypes and TNM stage. (**D**) The enrichment scores of 22 kinds of immune cells across the four subtypes.

Based on the expression profile of 360 NRLs, we categorized the patients with BC into various groups using the NMF consensus clustering. The optimal clustering number was identified when the k value was 4. Subsequently, we applied four molecular subtypes, consisting of 530 cases in cluster 1, 184 cases in cluster 2, 156 cases in cluster 3, and 67 cases in cluster 4 (Fig. 1A). KM analysis indicated that patients in cluster C1 had a poor OS than those in the other three clusters (p = 0.008, Fig. 1B). In addition, a significant difference in the expression of NRLs and TNM stage between the two subtypes was observed (Fig. 1C). The close correlation between our NRLs subtypes and clinical traits further illustrates the accuracy and stability of our identification of NRLs patterns in BC.

### Immune activities of the molecular subtypes

To understand the characteristics of immune cells in different subtypes, we conducted the distinction in 22 kinds of immune cell infiltration between the four subtypes (Table S3). Among the TIICs, activated memory CD4 ( +) T cells, CD8 ( +) T cells, resting and activated dendritic cells, M1 macrophages, activated NK cells, plasma cells, T cells gamma delta were significantly low infiltration in subtype1, and M2 macrophages was significant enrichment of in subtype1 group (Fig. 1D).

### Establishment of NRLs signature

**Figure 2:**
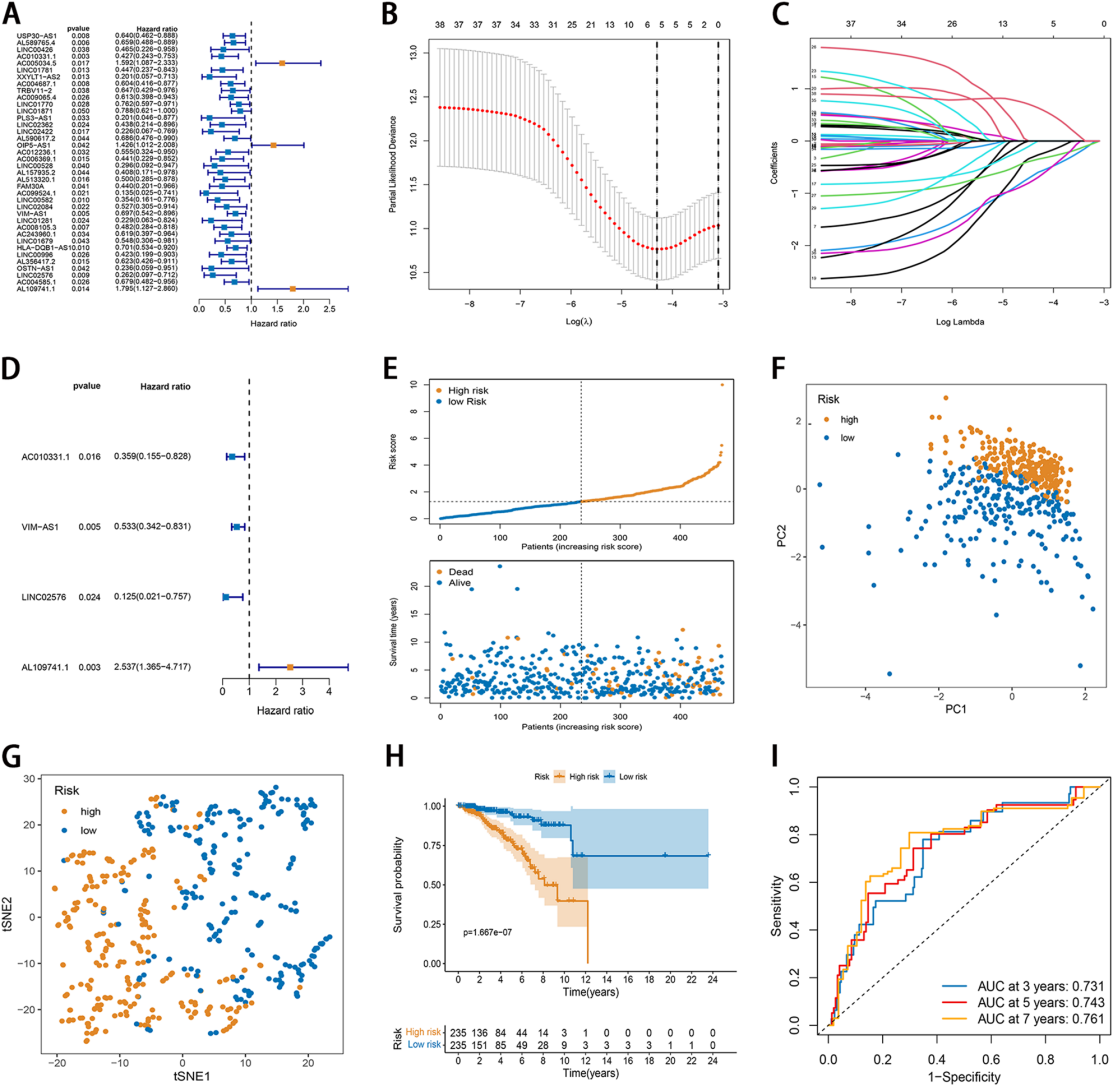
Development of necroptosis-related lncRNA signature in the training set. (**A**) The NRGs closely related to the prognosis of breast cancer patients were screened by univariate Cox regression analysis. (**B**) Regression coefficient profiles of identified NRLs in the TCGA cohort. (**C**) Ten-time cross-validation for tuning parameter selection. (**D**) Forrest plot showed that a total of 4 NRLs were identified as prognosis-related by multivariate cox analysis. (**E**) The distribution and value of the risk scores. (**F**) PCA of breast cancer patients according to the risk score. (**G**) t-SNE of breast cancer patients according to the risk score. (**H**) Kaplan–Meier curves for OS in high- and low-risk groups. (**I**) ROC curve analysis shows the predictive efficiency of the established signature.

The entire dataset was randomly categorized into the training cohort (n = 470) and testing cohort (n = 467) at a ratio of 1:1. The univariate Cox regression performed on 360 NRLs revealed NRLs to be relevant to the OS of BC patients (p < 0.05; Fig. 2A). Subsequently, five NRLs were identified after LASSO regression (Fig. 2B,C). Finally, multivariate Cox regression revealed four FRLs (AC010331.1, VIM-AS1, LINC02576, and AL109741.1; Fig. 2D). The risk score was calculated using the following formula: risk score = (− 1.0254 × Exp AC010331.1) + (− 0.6291 × ExpVIM-AS1) + (− 2.0802 × ExpLINC02576) + (0.9311 × AL109741.1). Among four lncRNAs, AL109741.1 was a risk factor for BC, while three lncRNAs (AC010331.1, VIM-AS1, and LINC02576) were protective factors for BC. The samples were assigned to the low- and high-risk subgroups according to the medium risk score. High-risk cases had elevated mortality and reduced survival time in comparison with low-risk counterparts (Fig. 2E). PCA and t-SNE both demonstrated individuals with distinct risk levels were distributed into two clearly delineated clusters (Fig. 2F,G). Kaplan Meier survival analyses revealed that OS in the high-risk subgroup was poorer than that in the low-risk subgroup (p < 0.001, Fig. 2H). The AUCs for 3-, 5- and 7-year survival were 0.731, 0.743, and 0.761, respectively (Fig. 2I). The C-index for 3-, 5- and 7-year survival was 0.714, 0.718, and 0.721, respectively (Supplementary Fig. 1A). Furthermore, we compared the ROC values of the risk score with other gene signatures in BC. As shown in Supplementary Fig. 2, the AUC of risk score was the largest compared to other gene signatures, demonstrating better predictive performance.

**Figure 3:**
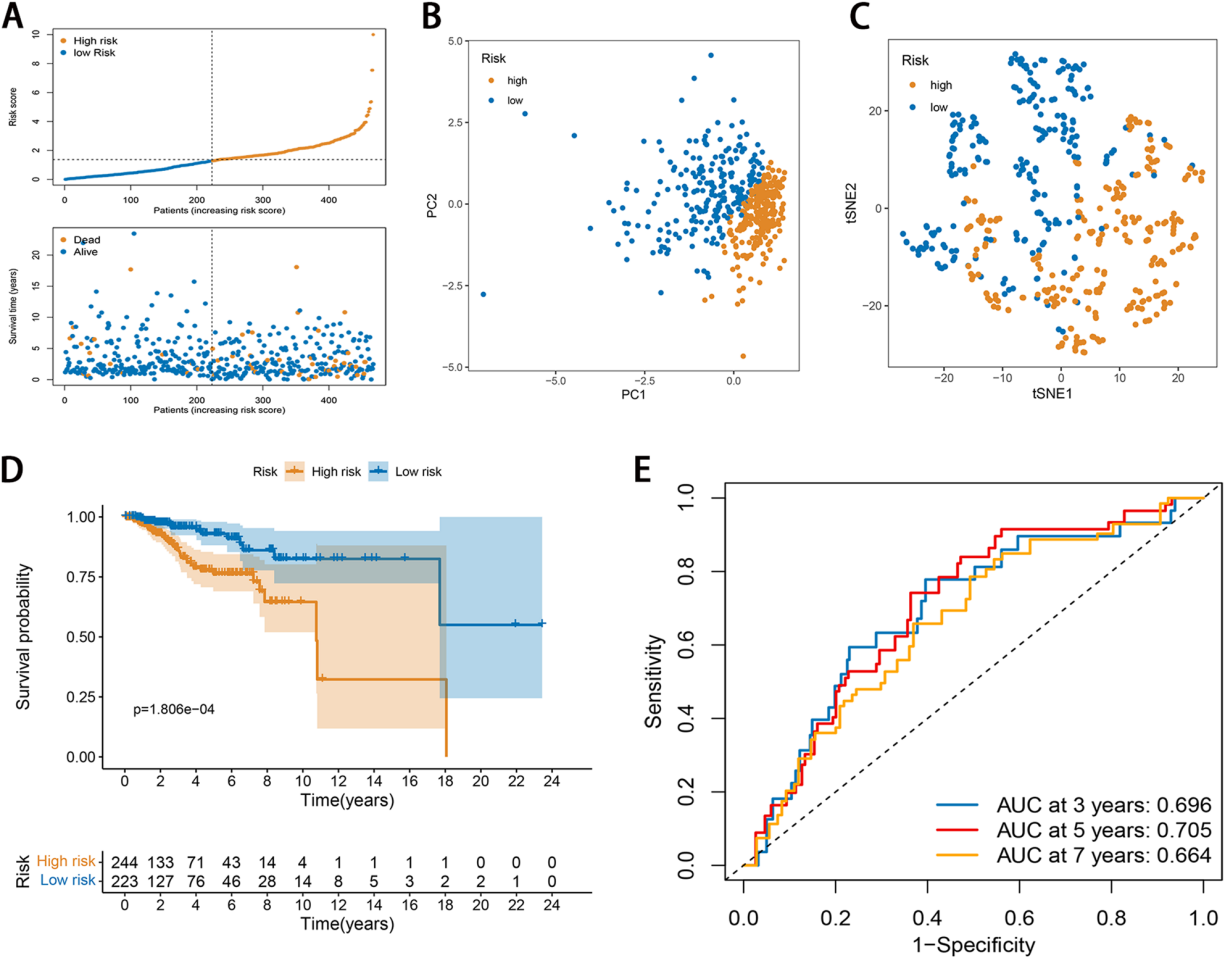
Evaluation of the signature in the testing set. (**A**) The distribution and value of the risk scores. (**B**) PCA of breast cancer patients according to the risk score. (**C**) t-SNE of breast cancer patients according to the risk score. (**D**) Kaplan–Meier curves for OS in high- and low-risk groups. (**E**) ROC curve analysis shows the predictive efficiency of the established risk score.

We also validated the signature for its accuracy in the testing set. The risk score of the prognostic signature for each patient was calculated using the same formula. Low-risk patients had prolonged survival and reduced mortality compared with high-risk counterparts (Fig. 3A). Both PCA and t-SNE demonstrated overt separation of both subgroups (Fig. 3B,C). The OS rate of the high-risk group was worse than that of the low-risk group (Fig. 3D). The ROC curves and C-index demonstrated that the signature harbored a promising performance to predict OS of BC in the testing set (Fig. 3E and Supplementary Fig. 1B).

### Clinical correlation analysis and stratification analysis of signature

**Figure 4:**
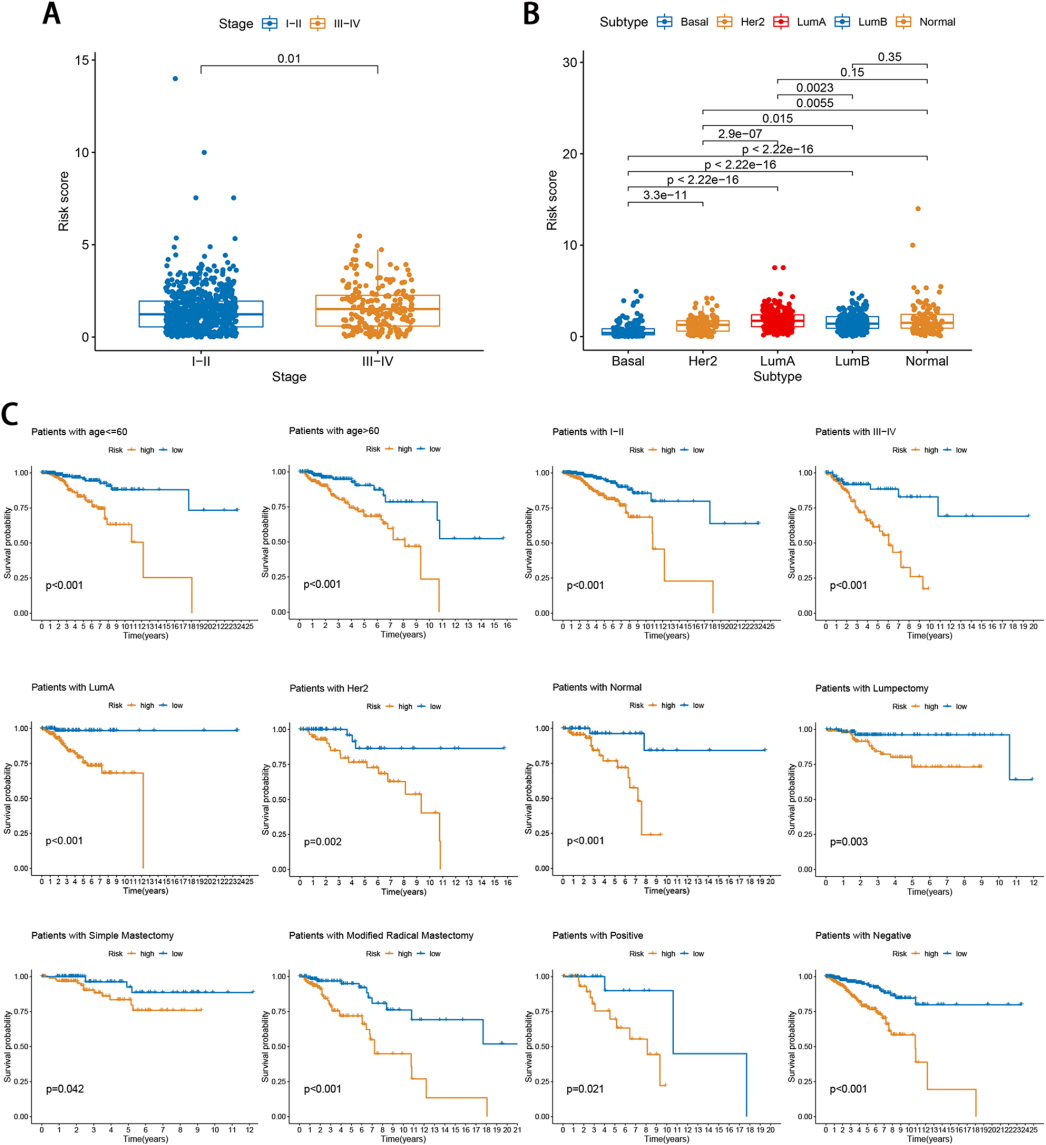
Clinical correlation and stratification analyses of signature. (**A**,**B**) The relationship between the signature, tumor stage, and molecular subtype. (**C**) Survival rates of the high- and low-risk patients in the subgroups are based on clinicopathological characteristics.

We explored the relationship between the signature and the clinical characteristics. The risk scores in the stage III-IV and four molecular subtypes (luminal A, luminal B, HER2, and normal) subgroups were significantly higher than those in the stage I-II and basal subtype subgroups (p < 0.05; Fig. 4A,B). To confirm the prognostic discriminatory power of the signature, we performed stratified survival analysis in various clinical subgroups. As the result shown in Fig. 4C, the OS of the low-risk patients based on age (p < 0.001), stage (p < 0.001), molecular subtype (p < 0.001 in luminal A, p = 0.002 in HER2, and p < 0.001 in normal), surgery type (p = 0.003 in lumpectomy, p = 0.042 in simple mastectomy, p < 0.001 in modified radical mastectomy), margin status (p = 0.021 in positive and p < 0.001 in negative) was significantly higher than that of high-risk patients.

### Immune infiltration and IPS analyses

**Figure 5:**
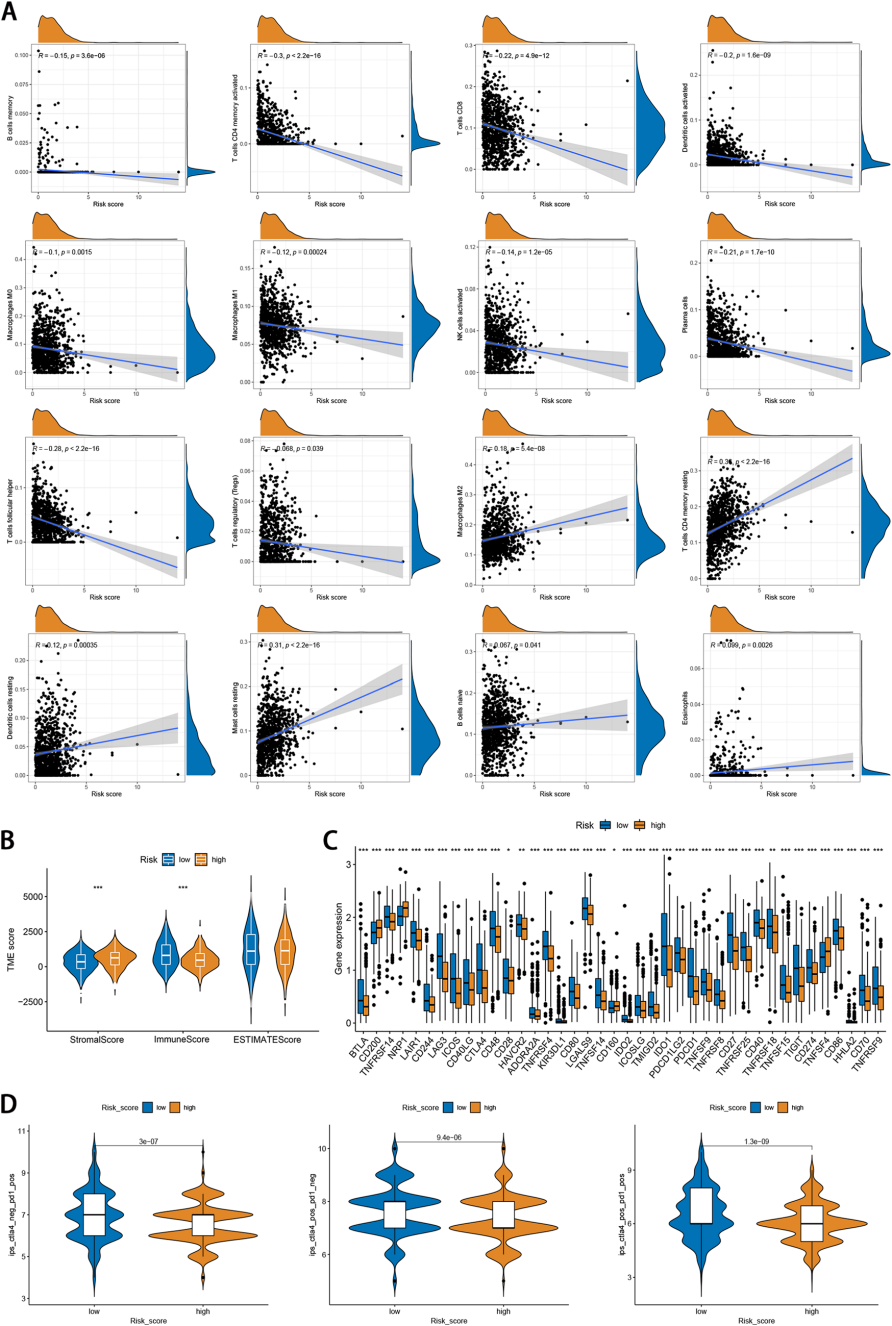
Immune landscape between high- and low-risk groups. (**A**) The infiltration levels of 22 immune cell types in the two risk groups. The spearman correlation coefficient R represents the degree and direction of the correlation between risk score and immune cell types. A positive R-value indicates a positive correlation, a negative R-value indicates a negative correlation, and an absolute value of R equal to zero indicates a zero correlation. (**B**) The relationship between signature and the TME score. (**C**) Difference of immune checkpoint expression between high- and low-risk groups. (**D**) Difference of immunogenicity between high- and low-risk groups.

We investigated the correlation between the risk score and the enrichment scores of 22 TIICs. High-risk score was negatively associated with infiltration of TIICs, including memory B cells, activated memory CD4 ( +) T cells, CD8 ( +) T cells, activated dendritic cells, M0 and M1 macrophages, activated NK cells, plasma cells, T follicular helper (Tfh) cells, Tregs, and positively associated with infiltration of M2 macrophages, resting memory CD4 ( +) T cells, resting dendritic cells, resting mast cells, naive B cells, and eosinophils (Fig. 5A). Furthermore, we observed that the risk score had a strong negative correlation with the TME scores (Fig. 5B). Immune checkpoint blocking has shown remarkable efficacy in the treatment of various types of cancer. We selected 47 types of immune checkpoints to analyze their expression differences between the two risk subgroups and found that the expression level of 36 immune checkpoints was elevated in the low-risk subgroup (Fig. 5C). Furthermore, the immunogenicity of two risk groups was analyzed by IPS analysis. The ips_ctla4_neg_pd1_pos, ips_ctla4_pos_pd1_neg, and ips_ctla4_pos_pd1_pos scores were higher in the low-risk group (Fig. 5D), suggesting that low-risk patients have a better response for immunotherapy.

### Somatic mutation landscapes and drug sensitivity analyses

**Figure 6:**
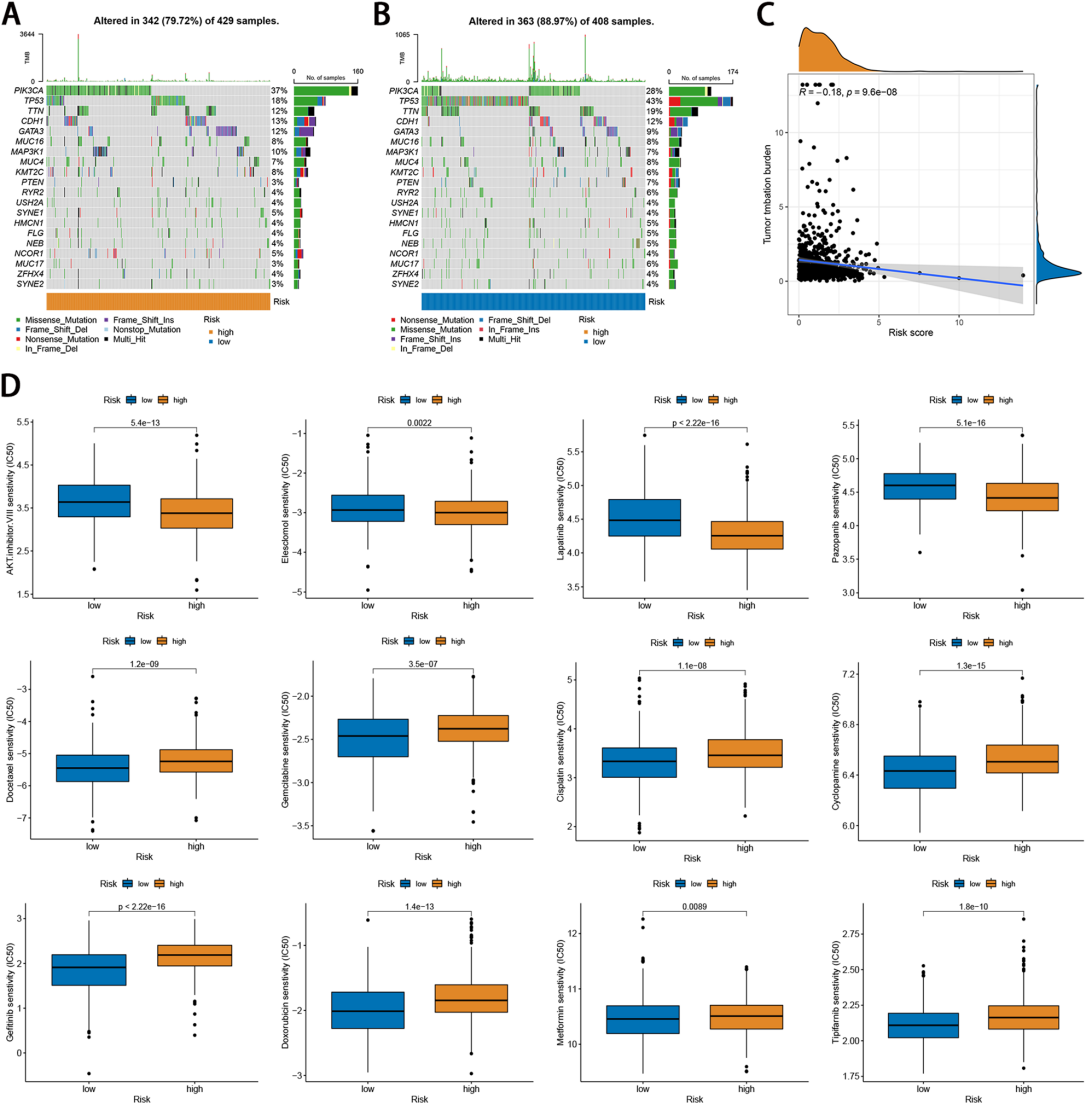
Somatic mutation landscapes and drug sensitivity analyses between high- and low-risk groups. (**A**,**B**) Waterfall diagram displays the mutation landscape of the top 20 most commonly mutation genes in the high- and low-risk groups. (**C**) Correlation of TMB with a risk score. (**D**) The IC50 values of four chemotherapeutic drugs in the high- and low-risk groups.

To assess the mutation landscape between two risk groups, we analyzed available somatic mutation data from the TCGA cohort. Among the two groups, the low-risk group had a higher mutation rate (89.97%) than that of the low-risk group (79.72%; Fig. 6A,B). The top five mutated genes in the two risk subgroups were PIK3CA, TP53, TTN, CDH1, and GATA3. Subsequently, we explored the relationship of the risk score with the TMB and observed that the risk score was negatively associated with TMB (Fig. 6C). In addition, we compared the sensitivity of two risk groups to common anticancer drugs to identify potential BC treatment modalities. As shown in Fig. 6D, patients in the high-risk group were more sensitive to AKT.inhibitor.VIII, Elesclomol, Lapatinib, Pazopanib, while those in the low-risk group were more sensitive to Docetaxel, Gemcitabine, Cisplatin, Cyclopamine, Gefitinib, Doxorubicin, Metformin, and Tipifarnib (Fig. 6D).

### Construction of prognostic nomogram

**Figure 7:**
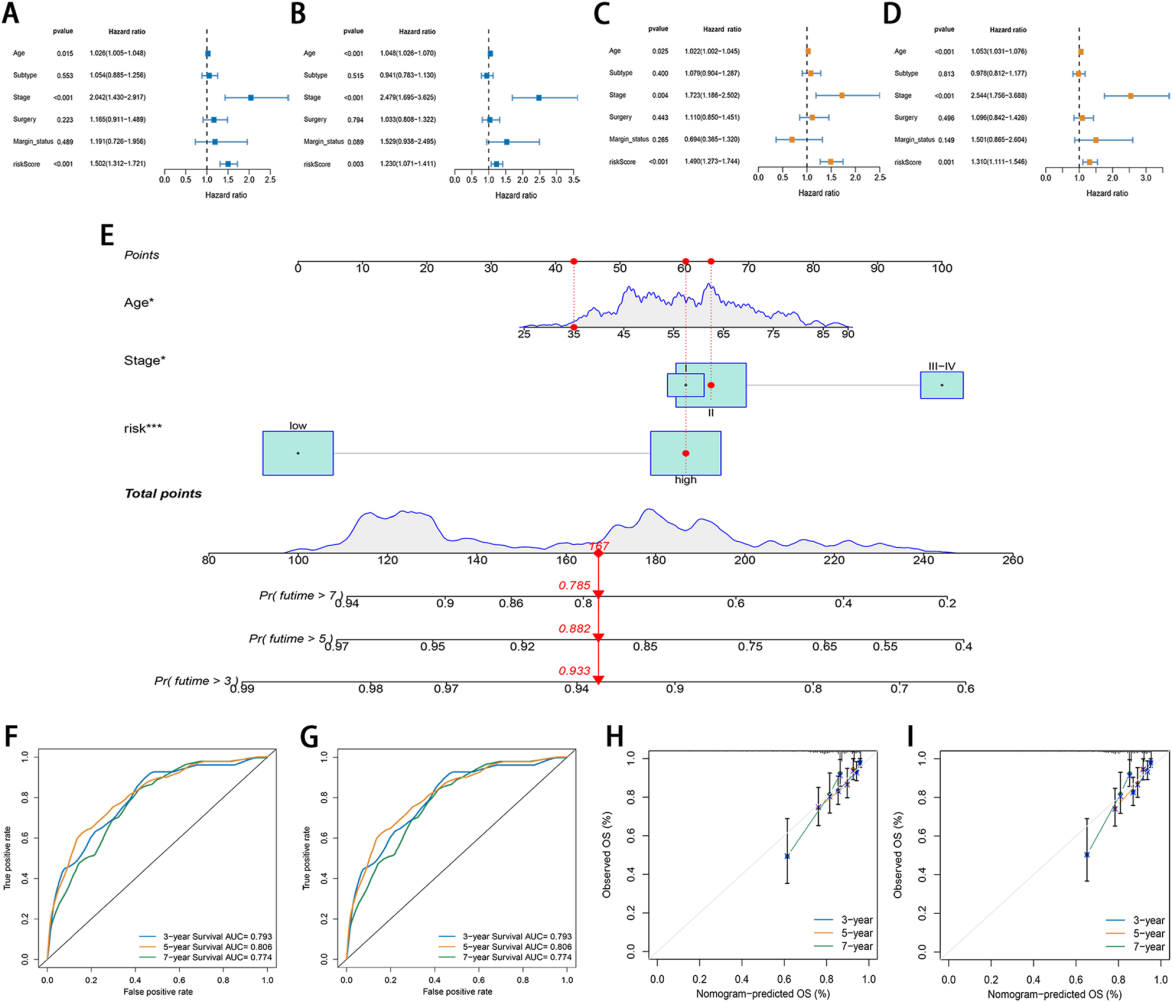
Construction and validation of the nomogram. (**A**,**B**) Univariate and Cox regression showed that the age, stage, and risk score were associated with OS in both sets. (**C**,**D**) Multivariate Cox regression shows that the age, stage, and risk score were independent prognostic indicators of OS in patients with breast cancer. (**E**) The nomogram combines risk signature and clinicopathological factors. (**F**,**G**) ROC curves for predicting the 3-, 5-, and 7-year ROC curves in the training and testing sets. (**H**,**I**) Calibration curves corrected for deviations in an agreement between the predicted and observed OS rates at 3, 5, and 7 years in both sets.

Univariable Cox regression analysis revealed age, stage, and risk score were associated with poor survival in both the training and testing cohorts (Fig. 7A,B). In multivariable analysis, after adjustment for other confounders, age, stage, and risk score remained an independent indicators in both sets (Fig. 7C,D). Based on multivariate Cox regression, the indicators, age, stage, and risk score, were included in the construction of the nomogram. Total scores were obtained by adding the individual indicator scores for age and risk score and predicting the probability of survival at 3, 5, and 7 years (Fig. 7E). Regarding the training set, the AUCs of the nomogram for the 3-, 5-, and 7- year OS predictions were 0.793, 0.806, and 0.774, respectively (Fig. 7F), whereas the AUCs of the testing set for predicting the 3-, 5-, and 7- year OS rates were 0.79, 0.736, and 0.776, respectively (Fig. 7G). Calibration curves showed that predicted survival times at 3, 5, and 7 years were consistent compared to the reference line in both training and testing sets (Fig. 7H,I), which showed that the nomogram was precise and stable.

### Functional analysis

**Figure 8:**
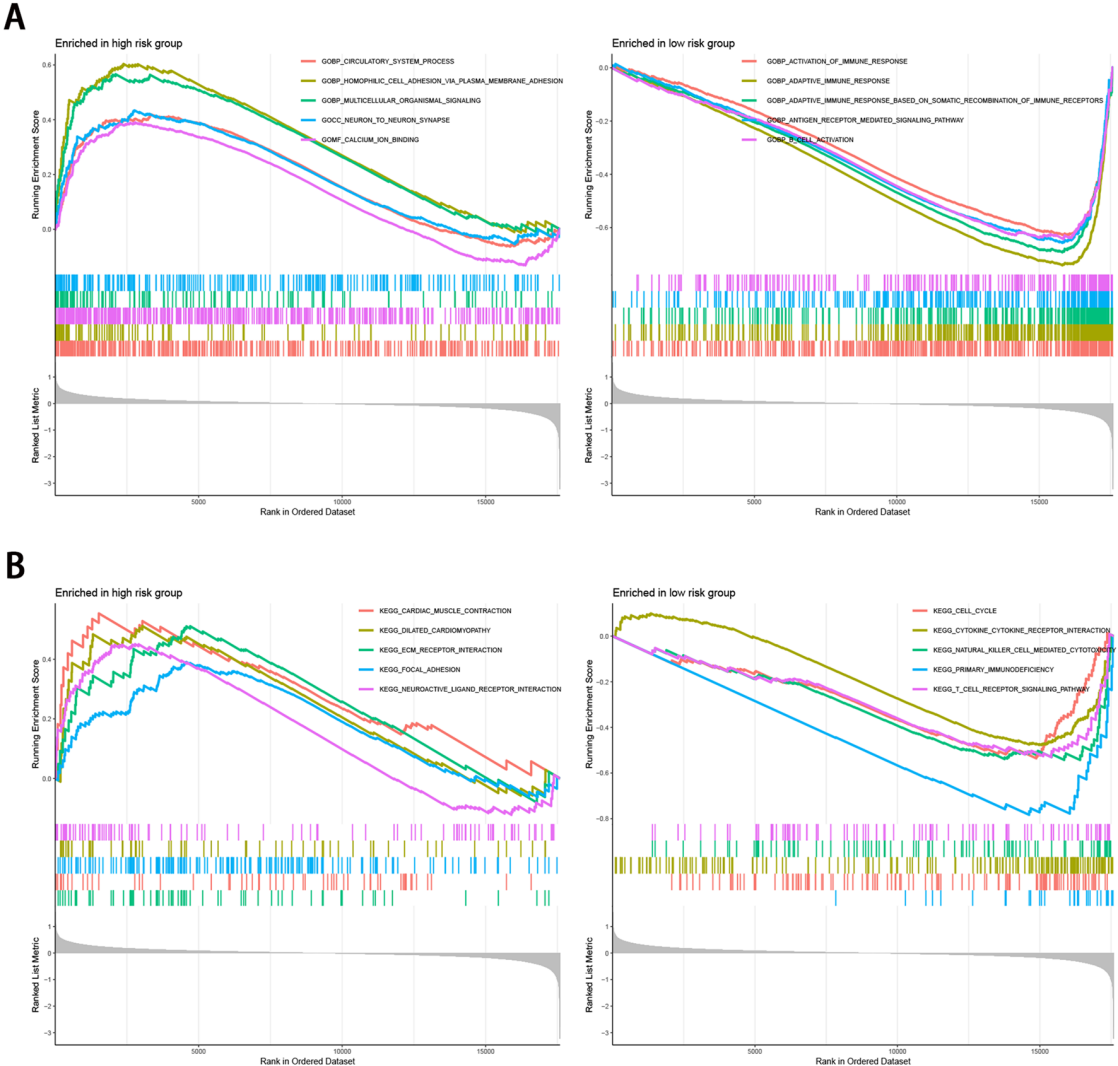
GSEA between high- and low-risk sets. (**A**) Representative results of enriched GO terms in two sets. (**B**) Representative results of enriched KEGG terms in two sets.

To investigate the potential biological pathways of the NRLs based risk score playing a role in BC, we conducted GSEA between the low- and high-risk subgroups using the entire gene network based on the TCGA dataset. The biological process mostly included activation of the immune response, adaptive immune response, antigen receptor mediated signaling pathway, and B cell activation in the low-risk group (Fig. 8A; Table S4). KEGG analysis revealed that gene sets associated with the low-risk group were significantly enriched in immune signaling pathways, such as cell cycle, cytokine cytokine receptor interaction, chemokine signaling pathway, primary immunodeficiency, and T cell receptor signaling pathway (Fig. 8B; Table S4).

## Discussion

Cell death is the natural endpoint of normal cell physiology and is necessary for mammalian growth and development, maintenance of biological homeostasis, and prevention of excessive proliferation of malignant cells. Necroptosis is a new form of programmed necrotic cell death that is mechanistically similar to apoptosis and morphologically similar to necrosis^6^. The necroptosis is regulated by distinct proteins, including RIPK1, RIPK3, and MLKL, and is characterized to be inhibited by the necrostatin-1^22^. Due to the inherent or acquired caspase-dependent apoptosis resistance of cancer cells, the therapeutic effect of antitumor drugs has been far from satisfactory. The resistance to apoptosis induced by anticancer agents is a hallmark of cancer^23^, and resistance to apoptosis is to a large extent a major obstacle to chemotherapy failure during cancer treatment. Due to its apoptosis-independent nature, bypassing the apoptotic pathway to induce cancer cell death is expected to overcome the deficiencies of traditional apoptosis-inducing chemotherapeutics. Moreover, tumor cells undergoing necroptosis can trigger robust antitumor immunity in vivo and in vitro, and immune checkpoint inhibitors (ICIs) can synergistically enhance their efficacy, even in ICI-resistant tumors^24^. Hence, necroptosis induction may provide promising therapeutic prospects, especially for patients underwent drug resistance to traditional chemotherapy or immunotherapy. Although increasing evidence have indicated that necroptosis might play a crucial part in cancer progression, the profiling and correlative signaling pathways of necroptosis in breast cancer has not yet been clarified.

In the present study, taking advantage of high-throughput RNA-seq data and aided by previous publications for experimentally supported lncRNAs related to necroptosis, we established clinical prognostic models to predict survival outcomes of BC patients. First, we identified 360 NRLs by performing a Pearson correlation analysis between 67 necroptosis-related genes and the lncRNAs. We then detected four distinct molecular subtypes and determined that cluster 1 was significantly associated with advanced clinical traits and poor survival outcomes. The proportions of 22 TIICs were significantly different between the four subtypes. Next, the combined analysis of Cox and LASSO regression was applied to establish an NRLs signature. The signature showed good predictive performance and was validated in the testing set. Furthermore, stratified survival analysis in various clinical subgroups confirmed the robust prognostic discriminatory power of the signature. Analysis of immunocyte infiltration by signature as well as immunologic function showed significant differences in the two risk score subgroups. Similarly, analyses of clinicopathological features, prognosis, TIICs, TME, somatic mutations, and drug response demonstrated significant differences between the two risk subgroups. In addition, we found that low-risk patients exhibited higher levels of immune checkpoints and showed higher immunogenicity in IPS analysis, suggesting a better response to immunotherapy.

Nomogram is a two-dimensional diagram giving a computation of mathematical functions and allows the estimation of specific endpoints. It has been widely used in clinical practice for its intuitive visual presentation^25^. The nomogram provides an individualized estimate of survival rather than a group estimate. This tool can be useful to patients and health care providers for counseling patients and their families regarding treatment decisions, follow-up, and prognosis^26^. In present study, a nomogram was constructed based on age, tumor stage, and risk score to further improve the performance and facilitate the use of the NRLs signature. The nomogram enables patients and physicians to create a more individualized surveillance program for BC, thus improving the prognosis. Functional enrichment analysis indicated that gene sets associated with the low-risk group were significantly enriched in immune signaling pathways.

Host immune dysfunction is a major factor in carcinogenesis. Particularly, the TME dictates the infiltration of inflammatory and immune cells with complex functions, either regulating tumor cell proliferation or triggering chronic inflammation, thereby inducing tumor progression via immune-suppressive pathways^27^. Previous studies have shown crosstalk between cells undergoing necroptosis and the remodeling of the immune microenvironment^24^. Notably, we also revealed an association between two risk groups and the immune microenvironment. We found that a high-risk score was negatively associated with the infiltration of memory B cells. B cells participated in various immune responses^28,29^.

Accumulating evidence has shown that tumor-infiltrating B lymphocytes inhibit tumor progression by secreting immunoglobulins, promoting T cell response, and directly killing cancer cells^30,31^. The enrichment of B cells and tertiary lymphoid structure has been considered a predictor of survival and response to immune checkpoint blockade in melanoma^32^. Increasing evidence shows that T cells play an important role in the anti-cancer immune response. This corresponds to our result that patients with high risk contained fewer CD4 (+) T cells and CD8 (+) T cells in TME. This provides evidence that the prognostic signature might predict the efficacy of immunotherapy. Dendritic cells are believed to be greatly involved in tumor antigen presentation to T cells and in controlling antitumor immunity, which might effectively suppress cancer cell proliferation^33^. In this study, we revealed that infiltration of activated Dendritic cells was negatively associated with a risk score. Since immune checkpoint blockade targeting PD-1, PD-L1 and CTLA4 has become an effective therapy to activate anti-tumor immunity, the application of this strategy to BC is drawing more and more attention^34–37^. In our research, the expression levels of immune checkpoints other than CD200, NRP1, and TNFSF4 were observed significant up-regulation in the low-risk subgroup. Hence, patients with low risk have a better response to ICIs. In addition, we found that low-risk patients showed higher immunogenicity in IPS analysis, suggesting a better response to immunotherapy.

Four NRLs obtained in the risk model were AC010331.1, VIM-AS1, LINC02576, and AL109741.1. These NRLs could be prognostic marker molecules, potential markers for BC, and potential therapeutic targets. Among the four NRLs, LINC02576, and AL109741.1 have not been reported to date. VIM-AS1 RNA is a 1.8-kb ncRNA transcribed from a bi-directional promoter shared with vimentin (VIM) mRNA and positively regulates VIM expression^38^. VIM-AS1 has been reported to be elevated in tumor tissues, especially in metastatic tumor tissues^38–40^. Sun et al.^39^ indicated that VIM-AS1 expression was significantly elevated in gastric cancer, and was associated with advanced clinicopathological features and worse prognosis. Knockdown of VIM-AS1 can suppress cell proliferation, migration, invasion, and epithelial-mesenchymal transition (EMT) by inhibiting FDZ1 expression and the Wnt/β-catenin pathway. Similarly, the expression of VIM-AS1 was markedly elevated in bladder cancer, and VIM-AS1 acts as a sponge for miR-655 to promote bladder cancer cell metastasis by regulating EMT^40^. However, a recent study revealed that VIM-AS1 expression was significantly downregulated in BC, and its expression was associated with menopause at age^41^. In the present study, we also found that VIM-AS1 was a BC suppressor, which is inconsistent with the findings of previous studies. AC010331.1 was found to be an autophagy-related lncRNA and associated with OS of bladder cancer^42^.

## Conclusions

In this study, four molecular subtypes based on 360 NRLs were identified, and these four molecular subtypes could predict clinical features, prognosis, and TIICs. Then, a 4-NRLs signature and nomogram were established and validated its predictive capability of OS in breast cancer patients. The signature can effectively predict the prognosis, TME cell-infiltrating characteristics, somatic mutations, chemotherapeutic drug sensitivity, and immunotherapy responses. These findings highlight the potential clinical implications of NRLs, suggesting that necroptosis may be the potential therapeutic target for patients with breast cancer.

## Supplementary Information

Supplementary Information 1.

Supplementary Information 2.

## Data Availability

The patient datasets in this study can be found here: TCGA database (http://www.cancer.gov/tcga), which is publicly available.

## Abbreviations

NRLs: Necroptosis-related lncRNAs
BC: Breast cancer
NMF: Non-negative matrix factorization
TIICs: Tumor-infiltrating immune cells
RIPK1: Receptor-interacting protein kinases 1
MLKL: Mixed lineage kinase domain-like pseudokinase
IPS: Immunophenoscore
lncRNAs: Long non-coding RNAs
LASSO: Least absolute shrinkage and selection operator
ROC: Receiver operating characteristic
PCA: Principal component analysis
t-SNE: T-distributed stochastic neighbor embedding
TME: Tumor microenvironment
GTEx: Genotype-tissue expression
FPKM: Fragments perkilobase million
GSEA: Gene set enrichment analysis
ICIs: Immune checkpoint inhibitors
MAF: Mutation annotation format
