# Supplementary material for: Necroptosis-related lncRNA signatures determine prognosis in breast cancer patients
Source: Sci Rep. 2022 Jul 4;12:11268. doi: 10.1038/s41598-022-15209-3 (PMC9253018; doi:10.1038/s41598-022-15209-3)
Supplement: Supplementary file 1 — Supplementary Information 1. [file 41598_2022_15209_MOESM1_ESM.docx]

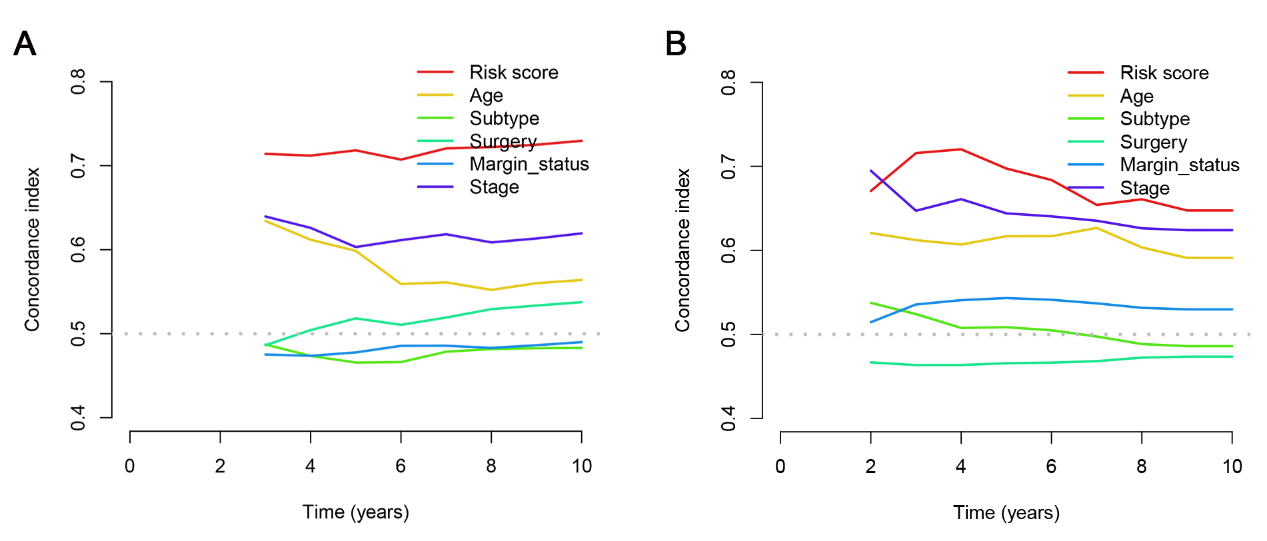


**Supplementary Figure 1** The C-index of the risk score in the training (A) and testing set (B).


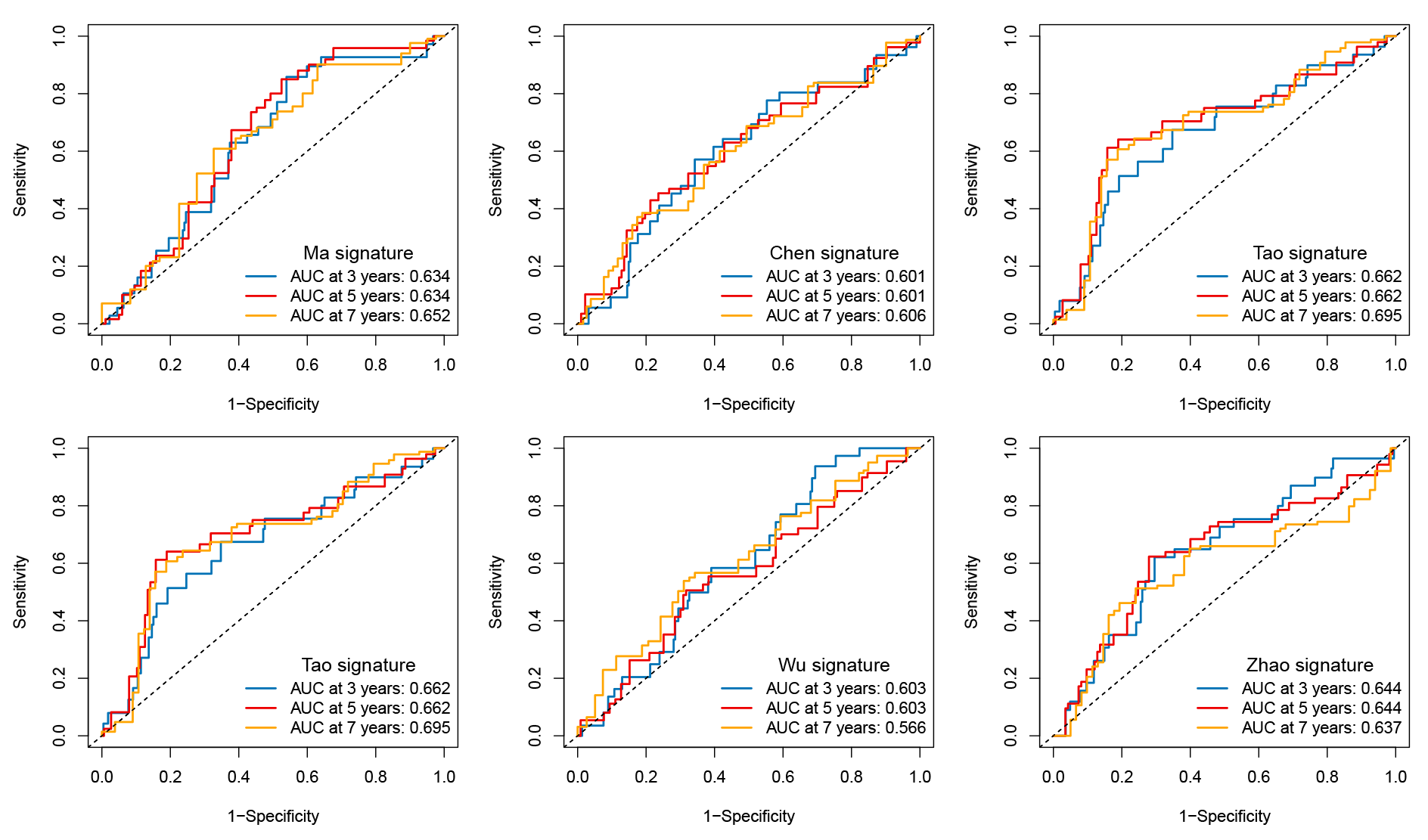


**Supplementary Figure 2** Comparison of ROC values for risk scores with other genetic signatures in BC.
